# On determining the sensitivity and specificity of a new diagnostic test through comparing its results against a non-gold-standard test

**DOI:** 10.11613/BM.2023.010101

**Published:** 2022-12-15

**Authors:** Farrokh Habibzadeh

**Affiliations:** Global Virus Network, Middle East Region, Shiraz, Iran

**Keywords:** biostatistics, diagnostic tests, prevalence, sensitivity and specificity

## Abstract

Diagnostic tests are important clinical tools. To assess the sensitivity and specificity of a new test, its results should be compared against a gold standard. However, the gold-standard test is not always available. Herein, I show that we can compare the new test against a well-established diagnostic test (not a gold-standard test, but with known sensitivity and specificity) and compute the sensitivity and specificity of the new test if we would have compared it against the gold-standard test. The technique presented is useful for situations where the gold standard is not readily available.

## Introduction

Diagnostic tests are among the important means commonly used in clinical medicine. Before a new test can be used in clinical practice, it should be evaluated for clinical validity. Studies assessing the clinical validity of a test (also termed diagnostic accuracy studies) involve determining the test performance indices including the test sensitivity (Se) and specificity (Sp) (*1*). Other common performance indices are positive and negative predictive values, and likelihood ratios, which can be calculated based on the Se and Sp and the prevalence (pr) of the disease of interest (*2*, *3*). To determine a test performance, its results should be evaluated against another test, the so-called reference standard (*4*). The reference standard can be a gold-standard test, *i.e.*, a test with a Se and Sp of 1.0 (or 100%). The gold-standard test can thus correctly discriminate those with and without the disease or condition of interest. For a test with binary results, the outcome is clear – positive or negative. For tests with continuous results, however, we need to set a cut-off value to categorize the results into positive or negative (*2*). Compared to the gold standard, the obtained results can be categorized into true-positive (TP), true-negative (TN), false-positive (FP), and false-negative (FN) results (Table 1a). The tests Se and Sp are defined as follows (*5*):



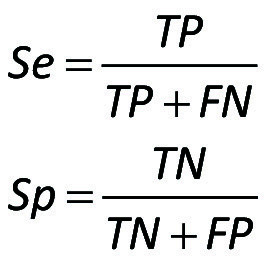



**Table 1:** Results of a hypothetical test validity study

**a**		**Gold-standard test**		
		Positive	Negative	**Total**
**T_1_**	Positive	TP: 85 π Se_1_	FP: 40 (1 – π)(1 – Sp_1_)	125
	Negative	FN: 15 π (1 – Se_1_)	TN: 360 (1 – π) Sp_1_	375
	**Total**	100	400	500
**b**		**T_1_**		
		Positive	Negative	**Total**
**T_2_**	Positive	107 pr Se_2,1_	104 (1 – pr)(1 – Sp_2,1_)	211
	Negative	43 pr (1 – Se_2,1_)	346 (1 – pr) Sp_2,1_	389
	**Total**	150	450	600
**c**		**Gold-standard test**		
		Positive	Negative	**Total**
**T_2_**	Positive	76 π Se_2_	64 (1 – π)(1 – Sp_2_)	140
	Negative	4 π (1 – Se_2_)	256 (1 – π) Sp_2_	260
	**Total**	80	320	400
**a**) a well-established test, T_1_, against the gold-standard test; **b**) a new test, T_2_, against T_1_; note that here, the true prevalence, π, is replaced by the apparent prevalence, pr (7) as T_1_ is not a gold standard; and **c**) another hypothetical study if T_2_ would have been tested against the gold standard. TP – True positive. FP – False positive. FN – False negative. TN – True negative. π – True prevalence. pr – Apparent prevalence. Sp – specificity. Se – sensitivity.				

Both the Se and Sp follow the binomial distribution. Then, the squared standard errors (SE^2^) for Se and Sp are:



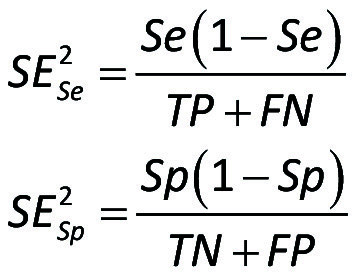



The prevalence of the disease (π), is then:



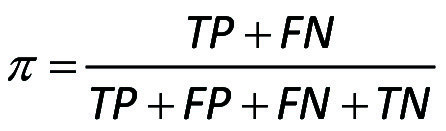



Combining Eq. 1 and Eq. 3, we have:



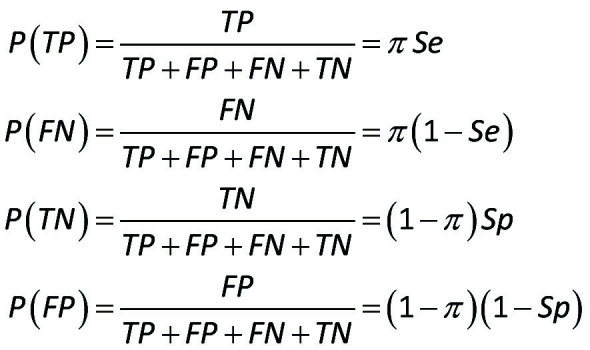



where P(x) designates the probability of x. To evaluate the Se and Sp of a new test, it is common to compare its test results against those obtained from a gold-standard test. Nonetheless, the gold-standard test may not always be available. It either does not exist or is very difficult or expensive to perform for certain disease conditions (*6*). The question arise is that whether it is possible to calculate the Se and Sp of the new test based on the results obtained from its comparison with a non-perfect reference standard – a well-established (but not a gold-standard) test? This is not a new question, and several solutions has so far been proposed (*1*). Herein, I wish to propose an analytical method to address the question raised.

## Stating the question

Suppose that we have a well-established test, say T_1_, with known Se and Sp (measured against a gold-standard test) of Se_1_ and Sp_1_ (Table 1a). Now, suppose that we have a new test, say T_2_, the results of which were compared against T_1_ (not against a gold standard), and that it had a Se and Sp (against T_1_) of Se_2,1_ and Sp_2,1_ (Table 1b). We wish to derive the Se and Sp of T_2_ (Se_2_ and Sp_2_), if it would have been tested against the gold standard (*e.g.*, Table 1c).

## The proposed solution

When we compare T_2_ against T_1_, the calculated prevalence, pr, is not really the true prevalence, π, as T_1_ is not a gold standard and thus would have FP and FN results. However, we can calculate the true prevalence, π, as follows (*7*):



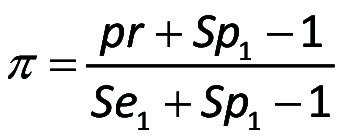



Based on Eq. 4 and basic probability rules, we have (Table 1) (*8*, *9*):



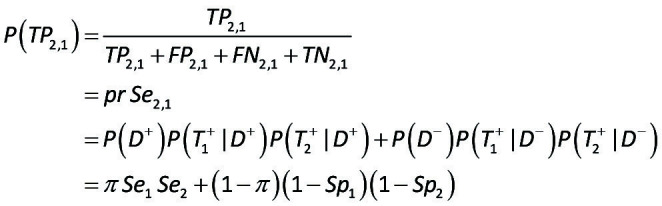



and



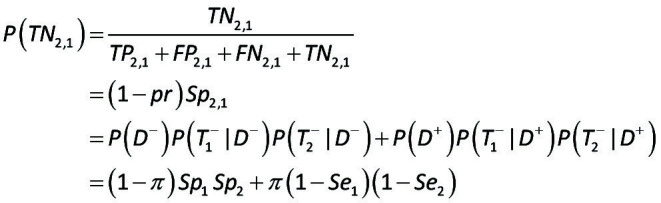



where T ^+^ and T ^–^ represent positive and negative test results; and D ^+^ and D ^–^, presence and absence of the disease, respectively. P(A|B) denotes the conditional probability of event A given event B.

Based on Eq. 6, we have:



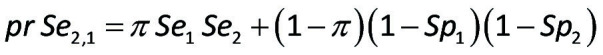



Solving for Se_2_, gives:



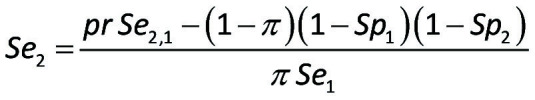



Based on Eq. 7, we have:



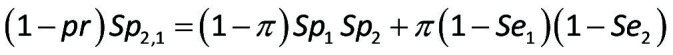



Then:



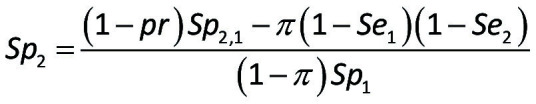



Equations 9 and 11 are a system of two simultaneous equations. Substituting π from Eq. 5 and solving for Se_2_ and Sp_2_, yield:



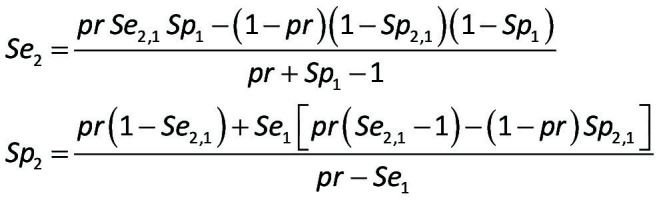



If f is a function of k independent random variables, then the squared SE of f can be calculated as (*10*, *11*):



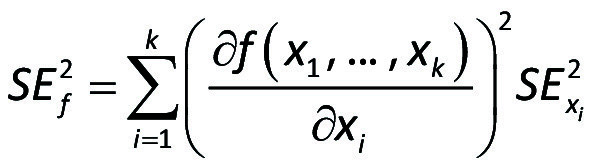



Assuming that Se_2_ is a function of independent random variables pr, Se_2,1_, Sp_2,1_, and Sp_1_ (Eq. 12), using Eq. 13 and employing basic calculus, we have:



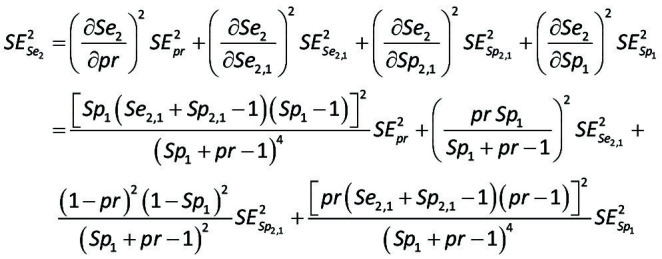



In the same way, assuming that Sp_2_ is a function of independent random variables pr, Se_2,1_, Sp_2,1_, and Se_1_ (Eq. 12), we have:



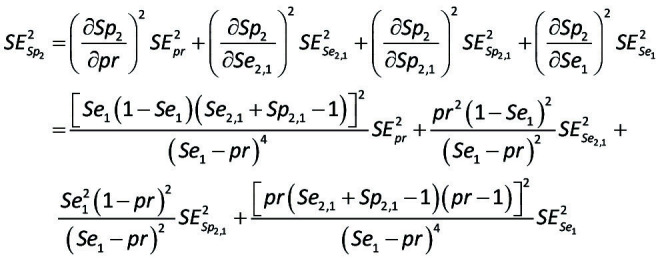



The SE for the Se and Sp of the tests can be calculated using Eq. 2.

## Discussion

It was shown that the test Se and Sp can be determined with acceptable accuracy even if the gold-standard test is not available. The Se and Sp of the new test (T_2_) derived by transforming the values obtained from its comparison with a non-gold-standard test (Se_2,1_ and Sp_2,1_) are acceptably close to the values if the test would have been compared with the gold-standard (Se_2_ and Sp_2_). The variances of the calculated Se_2_ and Sp_2_ (Eqs. 14 and 15) are higher than those you might obtain if you would have compared T_2_ directly against the gold standard, instead of T_1_. This is attributed to the uncertainty exist in the variables used for the calculation (Eq. 12). To examine the application of the technique proposed let us apply it to an example.

## Example

Suppose that in a validity study of 500 (arbitrary chosen) randomly selected people, a diagnostic test (let us call it T_1_) was tested against the gold standard (Table 1a), and that the test could correctly identify 85 of 100 diseased people, hence a Se (Se_1_) of 0.85, and 360 of 400 disease-free individuals, hence a Sp (Sp_1_) of 0.90 (Table 1a). The calculated SE^2^ for the Se_1_ and Sp_1_ are 1.3 × 10^-3^ and 2.3 × 10^-4^, respectively (using Eq. 2). Also, suppose that in a validity study on 600 (arbitrary chosen) randomly selected people, the results of a new diagnostic test, T_2_, was compared against T_1_ (Table 1b). Based on the information provided, the apparent prevalence, pr, is 0.25 (SE^2^ = 3.1 × 10^-4^). Using Eq. 5, the true prevalence (π) is:



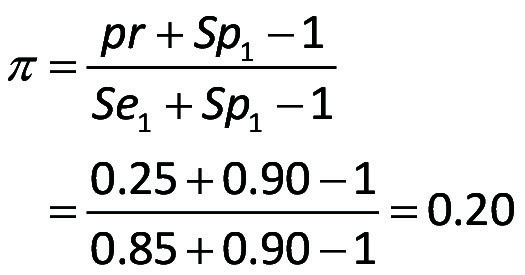



which is correct when the disease prevalence is measured by a gold-standard test (Table 1a). The Se and Sp (along with their SE^2^) of T_2_ against T_1_ (Table 1b), are then:



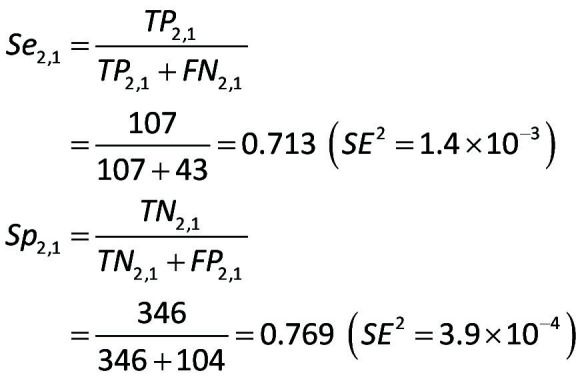



Plugging in the values in equations 12, 14 and 15, estimations of Se_2_ and Sp_2_ are 0.95 (SE^2^ = 8.0 × 10^-3^; 95% confidence interval (CI): 0.77 to 1.00) and 0.80 (SE^2^ = 5.4 × 10^-4^; 95% CI: 0.75 to 0.85), respectively, which are compatible with the results if T_2_ would have been compared against the gold-standard test – 0.95 (SE^2^ = 5.9 × 10^-4^; 95% CI: 0.90 to 1.00) and 0.80 (SE^2^ = 5.0 × 10^-4^; 95% CI: 0.76 to 0.84), respectively (Table 1c). Note that the 95% CI of the calculated Se_2_ and Sp_2_ when they are derived through comparing the results with T_1_ is wider than those if they are directly compared against a gold-standard test.

In conclusion, it seems that this technique is useful, particularly where the gold-standard test is not readily available or is expensive. Further studies are needed to elaborate on the conditions of the validity study where the Se_1_ and Sp_1_ are estimated, the minimum number of data points examined, the probable effect of the prevalence of the disease or condition of interest on the choice of the reference test, among other things.
